# Essentials of Materia Medica and Therapeutics

**Published:** 1865-09

**Authors:** 


### Books Reviewed.

Essentials of Materia Medica and Therapeutics. By Alfred Baring Garrod, M. D., F. R. S., etc New York: William Wood & Co., 1865.

The introductory chapter explains the meaning of the groups into which medicines are divided. The work proper begins with inorganic substances, and gives an accurate and detailed account of the natural history, physical and chemical properties, officinal preparation, therapeutical application and dose, with the adulterations to which they are frequently subjected.

The organic substances are treated of fully and concisely, stating when vegetables, roots, barks, flowers and fruits, intended for medical preparation should be gathered and how preserved. The last chapter is upon Test Solutions for quantitative and qualitative analyses of substances contained in the pharmacopœia, with an explanation of their more important applications. It will thus be observed that the book comprises a complete work upon materia medica. "*Essentials*" might very properly have been omitted in the title, thus not misleading into the idea that we were to have a work exclusively upon the truly essential articles of the materia medica.

No one will at present attempt to write a book upon the really valuable and thoroughly tested articles of medicine, since, in the first place they are too few in number; in the second there is too great discrepancy in opinion as to which are truly essential; and finally, if the best man in the world should write a book upon this subject, confining himself to what he knew of the action and value of medicine, he would not be tolorated at all; it would make too small a book, and would generally be regarded as containing nothing. In order to make a book upon materia medica, everybody's opinions must be published upon the action of all known substances, and their supposed influence upon disease. The book before us has many excellencies, and no faults which have led us into the above remarks, but we have often wished that we could really find a work upon the essentials of materia medica. We venture the opinion that no author has ever published any work, which in his own estimation merited such a name; the *un*-essentials of materia medica would be a very truthful and appropriate name for all these works, but they have only erred in leaving off the *un* in the title page, it is sufficiently obvious throughout the body of all our works upon the articles of medicine. The work by Garrod stands high in value as a scientific and complete work, It is perhaps unsurpassed in excellence; its title only afforded us a text for expressing a long cherished and carefully considered opinion, that a great majority of our medicinal substances, so-called, are the *un*essentials of materia medica.

